# Identification and Characterization of Mycemycin Biosynthetic Gene Clusters in *Streptomyces olivaceus* FXJ8.012 and *Streptomyces* sp. FXJ1.235

**DOI:** 10.3390/md16030098

**Published:** 2018-03-20

**Authors:** Fangying Song, Ning Liu, Minghao Liu, Yihua Chen, Ying Huang

**Affiliations:** 1 State Key Laboratory of Microbial Resources, Institute of Microbiology, Chinese Academy of Sciences, Beijing 100101, China; songfangying_@126.com (F.S.); fussliu@126.com (N.L.); lysf1987313@163.com (M.L.); chenyihua@im.ac.cn (Y.C.); 2 College of Life Sciences, University of Chinese Academy of Sciences, Beijing 100049, China

**Keywords:** mycemycin, dibenzoxazepinone, biosynthesis, gene cluster, pathway, *Streptomyces*

## Abstract

Mycemycins A–E are new members of the dibenzoxazepinone (DBP) family, derived from the *gntR* gene-disrupted deep sea strain *Streptomyces olivaceus* FXJ8.012Δ1741 and the soil strain *Streptomyces* sp. FXJ1.235. In this paper, we report the identification of the gene clusters and pathways’ inference for mycemycin biosynthesis in the two strains. Bioinformatics analyses of the genome sequences of *S. olivaceus* FXJ8.012Δ1741 and *S*. sp. FXJ1.235 predicted two divergent mycemycin gene clusters, *mym* and *mye*, respectively. Heterologous expression of the key enzyme genes of *mym* and genetic manipulation of *mye* as well as a feeding study in *S*. sp. FXJ1.235 confirmed the gene clusters and led to the proposed biosynthetic pathways for mycemycins. To the best of our knowledge, this is the first report on DBP biosynthetic gene clusters and pathways.

## 1. Introduction

Dibenzoxazepinones (DBPs) constitute an important family of heterocyclic compounds with two aromatic ring systems [1]. DBPs exhibit various bioactivities, including antitumor, antioxidant, anti-HIV reverse transcriptase, etc. [1,2], which have encouraged researchers to put considerable effort into the synthesis of DBP skeletons [3,4,5,6]. However, there have been few cases of either natural DBPs or their biosyntheses.

**Figure 1:**
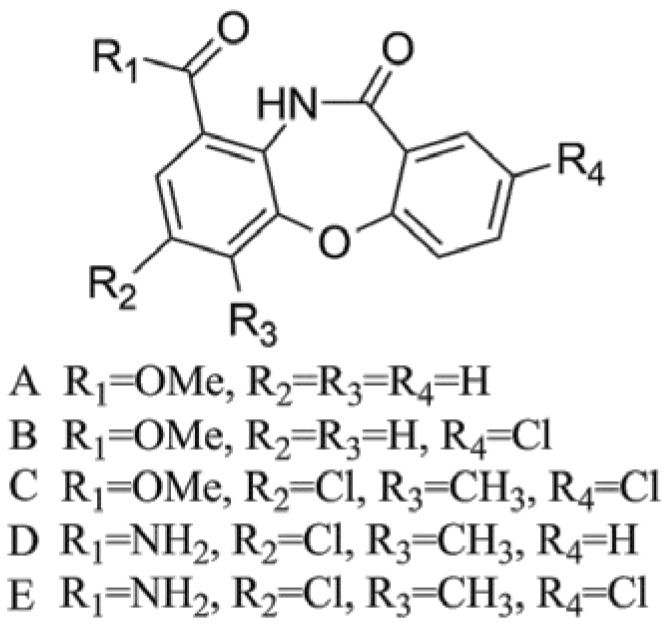
Structures of mycemycins A–E [7].

In our previous work, we reported mycemycins A–E (Figure 1) as new members of the DBP family with weak inhibitory activity on HIV-1 reverse transcriptase [7]. Mycemycins A and B were isolated from an acidic, red soil-derived strain *Streptomyces* sp. FXJ1.235 and mycemycins C–E from a *gntR* gene-disrupted deep sea strain, *Streptomyces olivaceus* FXJ8.012Δ1741. All the mycemycins are either mono- or bis-chlorinated derivatives except mycemycin A. Mycemycin B is mono-chlorinated at the R_4_ position, while mycemycin D is mono-chlorinated at the R_2_ position. Mycemycins C and E are chlorinated at both positions (Figure 1). These chemical structures indicate that different halogenation modifications might occur during mycemycin biosynthesis in *S. olivaceus* FXJ8.012Δ1741 and *S.* sp. FXJ1.235. To gain an understanding of the biosynthetic processes of DBPs, in this study, we identified mycemycin biosynthetic gene clusters and proposed biosynthetic pathways in the two different *Streptomyces* strains by genome analysis, heterologous expression, genetic manipulation, and precursor feeding. Our results show that, although they contain a series of homologs and code for similar pathways, the gene clusters responsible for mycemycin biosynthesis in the two strains are different in both arrangement and content. The marine-derived strain, *S. olivaceus* FXJ8.012Δ1741, possesses a continuous secondary biosynthetic gene cluster for mycemycins, while the soil-derived strain, *S.* sp. FXJ1.235, has a discontinuous mycemycin gene cluster that recruited two primary metabolic genes.

## 2. Results and Discussion

### 2.1. Sequence Analyses and Gene Organizations of the Mycemycin Biosynthetic Gene Clusters

**Figure 2:**
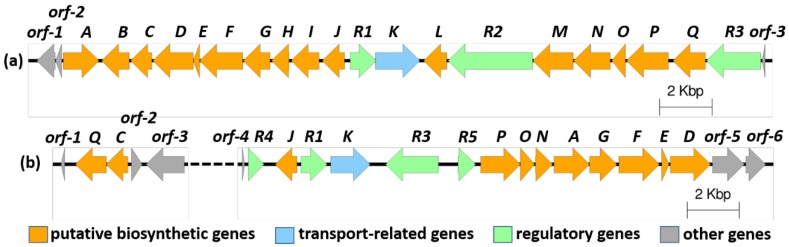
Organization of the mycemycin biosynthetic gene clusters in *S. olivaceus* FXJ8.012Δ1741 (**a**) and *S.* sp. FXJ1.235 (**b**). The dotted line represents a distance of >270 kb in the genome.

To find the putative gene clusters for mycemycins in *S. olivaceus* FXJ8.012Δ1741 and *S.* sp. FXJ1.235, we sequenced the genomic DNAs of the two strains and searched the draft genome sequences for halogenase-encoding genes. Two putative halogenase genes, *mymM* and *mymP*, were found in *S. olivaceus* FXJ8.012Δ1741, while only one candidate, *myeP*, was present in *S.* sp. FXJ1.235. Comparative sequence analyses of the proteins encoded by the genes flanking these putative halogenase genes revealed that they were likely involved in the biosynthesis of mycemycins. In *S. olivaceus* FXJ8.012Δ1741, the predicted gene cluster (*mym*) contained 20 open reading frames (ORFs) spanning a ~26.8 kb continuous region (Figure 2 and Table 1). However, in *S.* sp. FXJ1.235 the predicted cluster (*mye*) seemed truncated, containing only 14 ORFs in a continuous region with a rearranged gene order, compared to their counterparts in *mym* (Figure 2 and Table S1). Two important *mym* genes, *mymC* and *mymQ*, which encode the putative tryptophan 2,3-dioxygenase (TDO) and kynureninase (KYN), respectively, were missing in the truncated *mye* cluster. TDO has been reported to catalyze the conversion of tryptophan (Trp) to *N*-formylkynurenine (NFK) [8,9], while KYN is responsible for the hydrolytic cleavage of the side chain from both 3-hydroxykynurenine and kynurenine to produce 3-hydroxyanthranilate and anthranilic acid, respectively [10]. These two processes are not only essential during the primary metabolism of kynurenine but also are involved in the precursor supply of many antibiotic biosyntheses [11,12,13]. Accordingly, they are most likely to be indispensable for mycemycin biosynthesis. Additional searches of the genome sequences resulted in the identification of a copy of TDO- and KYN-encoding genes in *S.* sp. FXJ1.235 and an additional copy of both genes in *S. olivaceus* FXJ8.012Δ1741, which were located in different genome scaffolds from those encompassing the predicted mycemycin gene clusters*.* That is, the genome of *S. olivaceus* FXJ8.012Δ1741 carried two copies of both TDO- and KYN-encoding genes, with one copy (*mymC* and *mymQ*) located in the *mym* gene cluster. However, the genome of *S.* sp. FXJ1.235 contained only one copy of the two genes, *myeC* and *myeQ*, respectively. The *myeC* and *myeQ* genes were adjacent to each other and located at least 270 kb away from the partial *mye* cluster, according to their positions in the genome scaffolds. Therefore, we speculated that *myeC* and *myeQ* were responsible for both the biosynthesis of mycemycins and the primary metabolism process in *S.* sp. FXJ1.235.

### 2.2. Heterologous Expression of the Key Enzyme Genes of Mym and in Vitro Activity Assay

**Figure 3:**
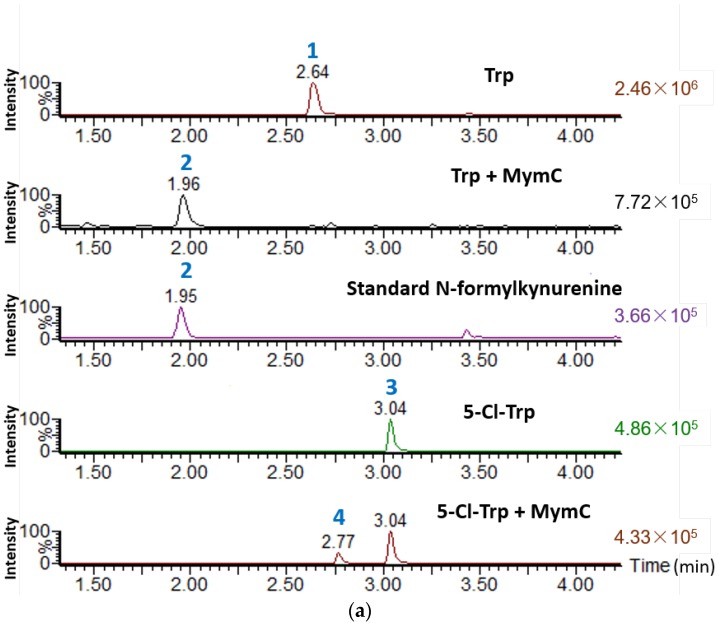

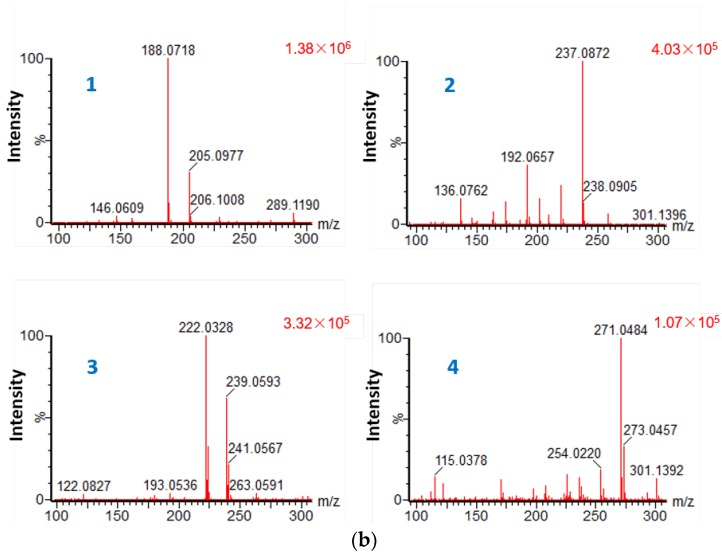
Ultra-high-performance liquid chromatography (UHPLC)-High-resolution mass spectrometry (HRMS) analyses (**a**) and High-resolution electrospray ionisation mass spectrometry (HR-ESI-MS) spectra (**b**) of the products from the in vitro enzymatic assay of MymC. 1: Trp; 2: *N*-formylkynurenine; 3: 5-Cl-Trp; 4: 5-Cl-*N*-formylkynurenine.

We found that the production of mycemycins C–E in FXJ8.012Δ1741 was unstable when performing genetic manipulation of the *mym* gene cluster. Thus, we carried out heterologous expression of the key enzyme genes of *mym* to verify their functions. According to the results of BLASTP [14] searching agains public databases, MymC showed a 59% identity to SibP, which was proposed to be a putative TDO during the biosynthesis of sibiromycin [15]. Previous studies have indicated that TDO is a member of heme-based dioxygenases and, in addition to catalyzing the conversion of Trp to NFK [9,16], it can also catalyze halogenated Trp, such as 6-fluoro-Trp [9]. To verify the function of MymC, in this study, we cloned and heterologously expressed *mymC* in *E. coli* rosetta (DE3). The resulting 35.2-KDa protein was then purified and subjected to an in vitro enzymatic activity assay using either L-Trp or L-5-Cl-Trp as a substrate. Ultra-high-performance liquid chromatography (UHPLC)-High-resolution mass spectrometry (HRMS) analysis of the enzymatic reaction products confirmed that MymC was able to convert not only L-Trp to NFK but also L-5-Cl-Trp to 5-Cl-NFK (Figure 3).

Unfortunately, heterologous expression of MymD, MymM, and MymP in *E. coli* failed despite the use of several strategies, including changing the specialized host strains, fusion protein technology, codon optimization, etc. MymD and MymM were expressed as inclusion bodies and MymP was not expressed in *E. coli*. Perhaps molecular chaperones or some uncertain culture conditions were essential for these recombinant proteins to fold properly.

### 2.3. Identification of the Mycemycin Biosynthetic Gene Cluster in S. sp. FXJ1.235

**Figure 4:**
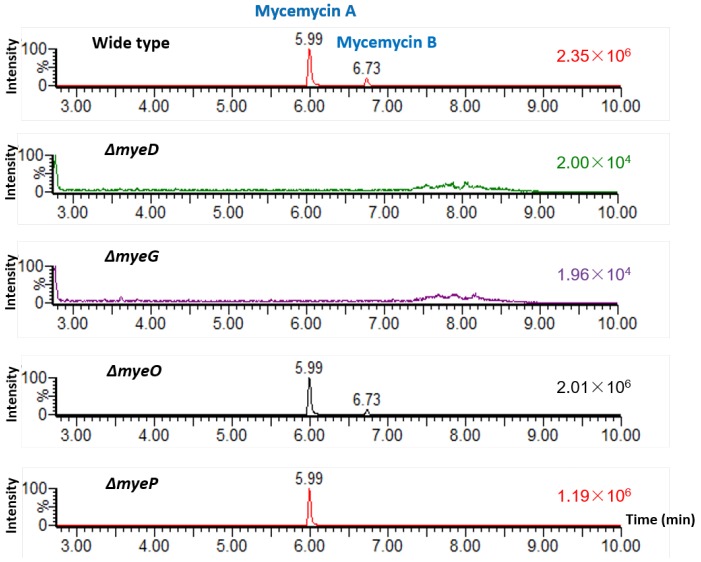
UHPLC-HRMS analyses of fermentation mycelium extracts of *S.* sp. FXJ1.235 wild type and its four mutants. The HR-ESI-MS spectra of mycemycins A and B are shown in Figure S1.

To verify that the predicted *mye* gene cluster was responsible for mycemycin biosynthesis in *S.* sp. FXJ1.235, the roles of *myeD*, *myeG*, *myeO*, and *myeP* were investigated by constructing four corresponding disruption mutants. For *myeD*, which encodes an amidohydrolase, a mutant Δ*myeD* containing a 1410 bp in-frame deletion within this gene was constructed. UHPLC-HRMS analysis of culture extracts showed that the production of both mycemycins A and B was abolished in this mutant (Figure 4). Disruption of *myeG*, which was predicted to encode a 3-oxoacyl-ACP synthase, was performed by replacing a 1045 bp fragment of this gene with the kanamycin resistance gene *neo* via a double-crossover homologous recombination. UHPLC-HRMS analysis also showed that the mutant Δ*myeG* no longer produced mycemycins (Figure 4). Inactivation of *myeP*, which encoded a 511-amino acid halogenase, was performed through the same knockout strategy as Δ*myeG*. This resulted in the abolishment of mono-halogenated mycemycin B production but did not interfere with the synthesis of the non-halogenated mycemycin A (Figure 4). These results confirmed that MyeD and MyeG were essential for mycemycin biosynthesis and MyeP was responsible for the post-halogenation of mycemycin in *S.* sp. FXJ1.235.

In addition, UHPLC-HRMS analysis of a mutant containing a 396 bp in-frame deletion within *myeO*, a putative flavin reductase gene, showed that neither mycemycin A nor B was abolished, indicating that *myeO* was not essential for mycemycin biosynthesis in *S.* sp. FXJ1.235 (Figure 4 and Figure S1).

MyeO and MyeP were proposed to form a two-component halogenase system [17,18]. In this system, the halogenation by MyeP needed the flavin reductase MyeO to catalyze the nicotinamide adenine dinucleotide-dependent reduction of flavin adenine dinucleotide which would provide the FADH_2_ for the halogenase [19]. However, several halometabolite biosynthesis gene clusters were found to be free of flavin reductase genes and the FADH_2_ needed may have been supplied by flavin reductases encoded elsewhere in the genome [18,20]. In *S.* sp. FXJ1.235, there were at least three putative flavin reductases encoded in the genome, and therefore, based on the result of the mutant Δ*myeO*, the other two flavin reductases might have supplied the FADH_2_ for halogenation during mycemycin B biosynthesis.

### 2.4. Feeding S. sp. FXJ1.235 with 5-Cl-Trp

**Figure 5:**
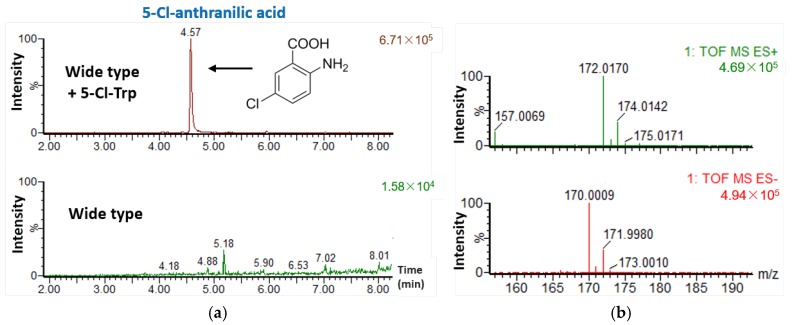
(**a**) UHPLC-HRMS analyses of fermentation liquid extracts of *S.* sp. FXJ1.235 feeding with 0.2 mM (final concentration) 5-Cl-Trp; (**b**) HR-ESI-MS spectra of the additional product 5-Cl-anthranilic acid in the feeding experiment.

To verify whether *S*. sp. FXJ1.235 could use 5-Cl-Trp to synthesize DBP dichloride derivatives similar to mycemycin C, 5-Cl-Trp was added to the fermentation medium. UHPLC-HRMS analysis showed no changes to the products in the resulting mycelium extracts but also identified an additional product in the fermentation liquid extracts (Figure 5a). However, the additional product was still mono-chlorinated. Its molecular formula was established as C_7_H_6_ClNO_2_ according to the [M + H]^+^ peak at *m*/*z* 172.0170 in HR-ESI-MS (Figure 5b) and the UV absorption at 208, 248, and 321 nm, respectively (Figure S2). Thus, this product was predicted to be 5-Cl-anthranilic acid; its chemical structure is shown in Figure 5a.

The structure of the additional product was very similar to that of anthranilic acid and was not hydroxylated. Considering the predicted functions of the *mye* genes, it is very likely that *S.* sp. FXJ1.235, 5-Cl-Trp can be converted to 5-Cl-anthranilic acid by successive enzymatic reactions of TDO (MyeC), amidohydrolase (MyeD), and KYN (MyeQ). However, the resulting 5-Cl-anthranilic acid could not be recognized by the subsequent enzymes responsible for the coupling of the anthranilic acid to the salicylic acid. This result suggested that some of the enzymes involved in the mycemycin biosynthesis in *S*. sp. FXJ1.235 had different substrate specificity than their counterparts in *S. olivaceus* FXJ8.012Δ1741.

### 2.5. Proposed Pathways for Mycemycin Biosynthesis

**Figure 6:**
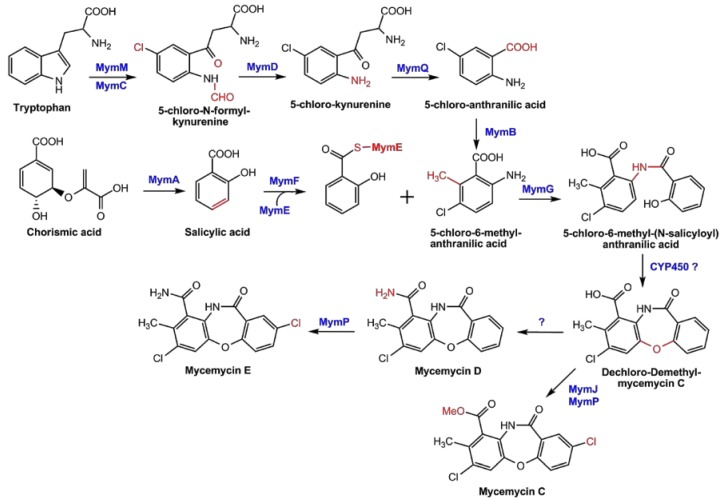
Proposed pathway for the biosynthesis of mycemycins C–E in *S. olivaceus* FXJ8.012Δ1741. The pathway for mycemycins A and B in *S.* sp. FXJ1.235 is shown in Figure S3.

Based on the results and analyses above, the biosynthetic pathways of mycemycin biosynthesis in *S. olivaceus* FXJ8.012Δ1741 and *S.* sp. FXJ1.235 are proposed and shown in Figure 6 and Figure S3, respectively. Initially, Trp was catalyzed by MymC/MyeC, a TDO, through the oxidative cleavage of the pyrrole ring and halogenated by MymM at the C-5 position of the benzene ring, resulting in NFK in *S.* sp. FXJ1.235 and 5-Cl-NFK in *S. olivaceus* FXJ8.012Δ1741. The initial halogenation was absent during the biosynthesis of mycemycins A and B in *S.* sp. FXJ1.235, since their chemical structures lacked chlorine atoms at this position. This prediction is consistent with the fact that the *mye* gene cluster contained only one halogenase gene while the *mym* gene cluster contained two. The amido bond of 5-Cl-NFK/NFK was cleaved by the amidohydrolase MymD/MyeD, resulting in 5-Cl-kynurenine/kynurenine. The alanine side chain of the latter was cleaved by the putative kynureninase, MymQ/MyeQ, to form 5-Cl-anthranilic acid/anthranilic acid [10,15]. The putative methyltransferase MymB catalyzed methylation at the C-6 position of benzene ring to produce 5-Cl-6-methyl-anthranilic acid in *S. olivaceus* FXJ8.012Δ1741. This step of methylation was also absent for mycemycins A and B, as their chemical structures lacked methyl groups at the same position. Moreover, salicylic acid was generated from chroismic acid by MymA/MyeA, and was loaded onto the *N*′-terminal peptidyl carrier protein domain of MymE/MyeE to yield salicyl thioester [21,22]. Next, the unusual beta-ketoacyl-ACP synthase, MymG/MyeG, catalyzed the formation of an amide bond between 5-Cl-6-methyl-anthranilic acid/anthranilic acid and salicyl thioester to generate 5-Cl-6-methyl-(*N*-salicyloyl)anthranilic acid/*N*-salicyloyl anthranilic acid [23]. The ether moiety was formed by oxidative coupling, potentially resulting from a diradical on the salicylic phenol and ortho to the amide of the anthranilic ring. Such oxidative couplings of phenolic compounds to ethers are known in bacteria, catalyzed by cytochrome P450 (CYP450) enzymes [24,25]. As we did not find any CYP450 genes in the mycemycin gene clusters, this reaction might be catalyzed by one of the multiple CYP450s encoded elsewhere in the genomes. Subsequently, the intermediate in *S. olivaceus* FXJ8.012Δ1741 was aminated at the carboxyl group to yield mycemycin D and then halogenated by the putative halogenase MymP at the C-5 position of the other benzene ring to yielded mycemycin E, or was methylated by MymJ at the carboxyl group and halogenated by MymP to generate mycemycin C (Figure 6). In *S.* sp. FXJ1.235, methylation and halogenation followed, producing mycemycins A and B (Figure S3).

It is interesting that *S. olivaceus* FXJ8.012Δ1741 and *S.* sp. FXJ1.235 had similar proposed biosynthetic pathways for mycemycins even though they were derived from different habitats, belonged to different species, and possessed mycemycin biosynthetic gene clusters with quite different genomic arrangements. Combined with the revelation of the feeding experiment result, we concluded that the two strains had gone through different evolutionary processes in marine and terrestrial habitats, respectively, to acquire and shape their mycemycin biosynthesis systems.

Mycemycin biosynthesis is supplied with precursors or intermediates from the primary metabolism which is not rare during secondary metabolites biosynthesis in actinobacteria [26]. *S.* sp. FXJ1.235 even engages two primary metabolic enzymes, TDO and KYN, for mycemycin biosynthesis. Perhaps the crosstalk between primary and secondary metabolisms provides a unique advantage for antibiotic-producing strains to balance the excess of nutrient substances and survive in harsh, nutrient-limited environments [27,28].

## 3. Materials and Methods

### 3.1. Strains, Plasmids and Media

Bacterial strains, plasmids, and primers used in this study are listed in Tables S2 and S3 in the Supporting Information. Detailed information of *S. olivaceus* FXJ8.012Δ1741and *S*. sp. FXJ1.235 was reported previously [7]. *Escherichia coli* Top 10 was used for propagating plasmids and *E. coli* ET12567/pUZ8002 was used for conjugation between *E. coli* and streptomycetes.

*Streptomyces* strains were grown on glucose-yeast extract-malt extract (GYM) agar medium at 28 °C for sporulation. GYM medium with 1 g/L NaCl was used as the fermentation medium for FXJ1.235, while modified R2 medium was used for FXJ8.012Δ1741. The culture conditions for mycemycin production were generally as described previously [7]. In short, spore suspensions were inoculated in 250 mL shake flasks containing 100 mL liquid GYM medium, which were then incubated as seed culture on a rotary shaker at 220 rpm and 28 °C for two days. Then 1 mL seed medium culture was transferred into a 250 mL shake flasks containing 100 mL fermentation medium. The inoculated flasks were incubated under similar conditions for seven days. *E. coli* strains carrying plasmids for nucleic acid manipulation were cultured in liquid or agar LB medium. When necessary, antibiotics were added at the following concentrations: ampicillin at 100 μg/mL, apramycin or kanamycin at 50 μg/mL, chloramphenicol at 25 μg/mL, and nalidixic acid at 25 μg/mL.

### 3.2. Genome Sequencing and Annotation

Genomic DNAs were extracted according to standard approaches [29]. Genome sequencing of *S. olivaceus* FXJ8.012Δ1741 was performed using Illumina genomic analyzer (Beijing Genomics Institute, Shenzhen, China), resulting in a total of 8,326,611 bp of genome sequence distributed across 519 contigs (≥500 bp). The genome of *S*. sp. FXJ1.235 was sequenced using an Illumina Hiseq system (Novogene, Beijing, China), resulting in a total of 8,852,829 bp of genome sequence distributed across 48 contigs (≥600 bp). The sequences obtained were annotated using Glimmer 3.02 [30], GeneMarkS [31], and BlastP [14]. The DNA sequences of mycemycin gene clusters have been deposited in the GenBank database. The accession number for *mym* is MG837055 and the numbers for *mye* are MG837053 (part I: *myeC*, *myeQ*, and *orf-1*~*orf-3*) and MG837054 (part II: the other *mye* genes and *orf-4*~*orf-6*).

### 3.3. Heterologous Expression and In Vitro Activity Assay of the TDO MymC

The TDO-encoding gene *mymC* was amplified from the *S. olivaceus* FXJ8.012Δ1741 genome DNA with primer pairs *mymC-EF* and *mymC-ER*. The amplimer was digested by *Hin*dIII/*Eco*RI after purification by agarose gel electrophoresis and then ligated with *Hin*dIII/*Eco*RI–digested plasmid pET28a to generate pET28a::*mymC*. Further verification was performed by PCR with primer pairs *T7* and *T7ter* and sequencing of the resulting amplimers. The recombinant plasmid was then transferred into *E. coli* rosetta (DE3).

Protein expression was induced by 0.5 mM isopropyl-b-d-thiogalactopyranoside (IPTG) in an LB medium containing 0.05 mg/mL kanamycin and 0.1 mg/mL heme at 15 °C for 20 h. The harvested cell pellets were resuspended in binding buffer [20 mM Tris-HCl (pH 8.0), 100 mM NaCl, 20 mM imadazole, 1 mM PMSF (phenylmethanesulfonyl fluoride) and 10% glycerol at pH 8.0] and lysed by sonication on ice. The resulting lysate was clarified by centrifugation and the supernatant was purified through Ni^2+^ affinity chromatograph strategy using a HisTrap HP column (GE Healthcare, Piscataway, NJ, USA). In brief, the supernatant was loaded onto a 5 mL pre-equilibrated HisTrap HP column and the bound protein was eluted with a linear gradient of 20 to 500 mM imidazole. The protein was then loaded onto a PD-10 desalting column (GE Healthcare) to remove imidazole and eluted with a desalting buffer [20 mM Tris–HCl (pH 8.0), 100 mM NaCl and 10% (*w*/*v*) glycerol]. Purified proteins were analyzed on 12% SDS-PAGE and the protein concentration was determined using a Pierce™ BCA protein assay kit (Thermo Scientific, Waltham, MA, USA).

Analysis of the in vitro activity of MymC was carried out by detecting the product formation at 321 nm (ε = 3750 M^−1^ cm^−1^ for *N*-formylkynurenine) [16]. The in vitro assay reaction (200 µL) was performed at 28 °C for 2 h which comprised of 20 mM Tris-HCl (pH 7.5), 100 mM NaCl, 10 mM ascorbic acid, 40 mM substrate, and 25 µM MymC solution. Subsequently, the reaction was stopped by adding an equal volume of methanol and analyzed by UHPLC.

### 3.4. Mutant Construction and Confirmation

Nucleic acid manipulations for *E. coli* and streptomycetes were performed according to standard approaches [29,32].

The Δ*myeP* and Δ*myeG* mutants were constructed as follows. The kanamycin resistance gene *neo* was amplified from pUC119::*neo* using primers *neo-Bgl*II*-F* and *neo-Bgl*II*-R* (Table S4). Upstream and downstream regions of approximately 2 kb flanking *myeP* were amplified from *S*. sp. FXJ1.235 genomic DNA using primer pairs *myeP*-LF/*myeP*-LR and *myeP*-RF/*myeP*-RR. These amplimers were separated by agarose gel electrophoresis and purified from the gel. The resulting DNA fragments were mixed with *neo* gene and *EcoRV*-digested plasmid pKC1139 to generate pKC1139::*myeP*::*neo* using Gibson isothermal assembly [33]. The recombinant plasmid was subsequently introduced into *S*. sp. FXJ1.235 by conjugation via ET12567/pUZ8002. Spores of exconjugants were harvested and spread on MS agar [29] containing kanamycin. After incubation at 40 °C for three days, apramycin-sensitive and kanamycin-resistant colonies were identified and further confirmed as *myeP* disruption mutants (Δ*myeP*) by PCR with primer pairs *myeP*-F and *myeP*-R along with a sequencing of the resulting amplimers. The Δ*myeG* mutant was constructed and verified in an analogous manner.

The Δ*myeO* and Δ*myeD* mutants were constructed as described above with minor modification. For Δ*myeO*, a 396 bp in-frame deletion extending from position +95 to +490 within *myeO* was performed. As for Δ*myeD*, a 1410 bp fragment from +92 to +1501 within *myeD* was also knocked out through in-frame deletion. After a temperature-sensitive experiment, apramycin-sensitive colonies were selected and further confirmed as target gene disruption mutants by PCR with corresponding primer pairs (Table S4) and sequencing of the resulting amplimers.

The mutants and wild type were fermented as mentioned above and the mycelium was extracted with enthanol as described previously [7].

### 3.5. Feeding Experiments

*S*. sp. FXJ1.235 was precultured as mentioned above for the seed culture and 1 mL seed culture was transferred into a 250 mL shake flask containing 100 mL fermentation medium supplemented with 0.2 mM (final concentration) 5-Cl-Trp. After incubation under similar conditions for seven days, the culture broth was centrifugated. The resulting mycelium was extracted with enthanol as described previously [7] and the supernatant was extracted twice with equal volumes of ethyl acetate. The extracts were then concentrated to dryness under vacuum.

### 3.6. Ultra-High-Performance Liquid Chromatography-High-Resolution Mass Spectrometry

The extracts above were redissolved separately in methanol and passed through a 0.45 µm membrane. A 2 µL of sample was then injected into a Waters ACQUITY UPLC BEH C_18_ column (2.1 mm × 50 mm, 1.7 µm, 45 °C) connected to a Waters ACQUITY UPLC/Xevo G2 Qtof MS system (Waters Corporation, Milford, MA, USA) equipped with an electrospray source. The column was eluted as follows: 0 min − 95% A + 5% B, 10 min − 0% A + 100% B, where A was water containing 0.1% formic acid and B was acetonitrile. The full scan data were acquired in the positive ion mode from 50 to 1200 Da with a 0.2 s scan time using the following settings: capillary voltage 3.0 kV; de-solvation temperature 350 °C; sample cone voltage 35 V; extraction cone voltage 4 V; source temperature 120 °C; cone gas flow 50 L/h; and desolvation gas flow 800 L/h. The mass spectrometer was calibrated across the mass range of 50–1200 Da using a solution of sodium formate. Data were centroided and *m*/*z* values were corrected during acquisition using an external reference consisting of a 0.2 ng/mL solution of leucine enkephalin infused at a flow rate of 5 μL/min via a lockspray interface, generating a reference ion at 556.2771 Da ([M + H]^+^). The lockspray scan time was set at 0.5 s with an interval of 15 s and data were averaged over three scans.

## 4. Conclusions

In this study, we applied bioinformatics analyses, heterologous expression for *S. olivaceus* FXJ8.012Δ1741, genetic manipulation, and a feeding experiment in *S.* sp. FXJ1.235, leading to the identification of divergent mycemycin biosynthetic gene clusters and the detection of similar proposed biosynthetic pathways in the two strains. It is the first report of DBP biosynthetic gene clusters and pathways. These findings also establish a foundation for biosynthesis of DBP derivatives with desired bioactivities.

## Figures and Tables

**Table 1:** Genes and proteins involved in mycemycin biosynthesis and their putative functions in *S. olivaceus* FXJ8.012Δ1741.

Gene	Size (AA)	Protein Homolog and Origin (Identity/Similarity)	Homolog in *mye* (Identity/Similarity)	Proposed Function
*orf-1*	231	WP_011030904.1 (89/95); *Streptomyces coelicolor*	-	Hypothetical protein
*orf-2*	78	None predicted in NCBI	-	Unknown
*mymA*	454	SsfH, ADE34507.1 (55/68); *Streptomyces* sp. SF2575	*myeA* (53/66)	Salicylate synthase
*mymB*	351	SibL, ACN39735.1 (65/76); *Streptosporangium sibiricum*	-	Methyltransferase
*mymC*	269	SibP, ACN39739.1 (59/70); *Streptosporangium sibiricum*	*myeC* (38/53)	Tryptophan 2,3-dioxygenase (TDO)
*mymD*	513	EFL38513.1 (61/71); *Streptomyces griseoflavus* Tu4000	*myeD* (62/76)	Amidohydrolase
*mymE*	82	EsmD3, AFB35628.1 (48/61); *Streptomyces antibioticus*	*myeE* (49/63)	Phosphopantetheine-binding protein
*mymF*	547	PchD, NP_252918.1 (48/61); *Pseudomonas aeruginosa* PAO1	*myeF* (61/73)	2,3-dihydroxybenzoate-AMP ligase
*mymG*	339	BomK, ALE27503.1 (52/70); *Streptomyces* sp. NRRL 12068	*myeG* (60/76)	Beta-ketoacyl-ACP synthase (amide bond formation)
*mymH*	220	EFL35401.1 (49/64); *Streptomyces viridochromogenes* DSM 40736	-	Hypothetical protein
*mymI*	354	KUK37103.1 (61/75); *Thermacetogenium phaeum*	-	3-deoxy-7-phosphoheptulonate synthase
*mymJ*	281	SCE38941.1 (60/68); *Streptomyces* sp. PpalLS-921	*myeJ* (63/72)	SAM-dependent methyltransferase
*mymR1*	329	SBU95411.1 (75/86); *Streptomyces* sp. OspMP-M45	*myeR1* (75/85)	Lrp/AsnC family transcriptional regulator
*mymK*	563	CB02009_orf6, OKJ63402.1 (69/81); *Streptomyces* sp. CB02009	*myeK* (69/79)	Multidrug MFS transporter
*mymL*	294	Carboxylesterase NlhH, ASO22469.1 (50/61); *Actinoalloteichus* hoggarensis	-	Alpha/beta hydrolase
*mymR2*	1072	WP_033441976.1 (38/49); *Saccharothrix* sp. NRRL B-16314	-	SARP family transcriptional regulator
*mymM*	511	PyrH, OSY47217.1 (61/76); *Streptomyces platensis*	-	Tryptophan halogenase
*mymN*	464	SDU28343.1 (53/68); *Amycolatopsis keratiniphila*	*myeN* (61/74, query cover 56%)	Sodium/hydrogen exchanger family
*mymO*	175	KtzS, ABV56599.1 (59/67); *Kutzneria* sp. 744	*myeO* (61/69)	Flavin reductase
*mymP*	530	RebH, 4LU6_A (63/76); *Lechevalieria aerocolonigenes*	*myeP* (75/83)	Tryptophan halogenase
*mymQ*	411	KynU, NP_250770.1 (44/60); *Pseudomonas aeruginosa* PAO1	*myeQ* (51/64)	Kynureninase (KYN)
*mymR3*	691	SBU95407.1 (56/67); *Streptomyces* sp. OspMP-M45	*myeR3* (57/68)	SARP family transcriptional regulator
*orf-3*	38	None predicted in NCBI	-	Unknown

## Supplementary Materials

The following are available online at http://www.mdpi.com/1660-3397/16/3/98/s1, Table S1: Genes and proteins involved in mycemycin biosynthesis and their putative functions in *S*. sp. FXJ1.235; Table S2: Strains used in this study; Table S3: Plasmids used in this study; Table S4: Primers used in this study; Figure S1: HR-ESI-MS spectra of mycemycin A (**a**) and mycemycin B (**b**); Figure S2: UV spectrum of 5-Cl-anthranilic acid; Figure S3: Proposed pathway for the biosynthesis of mycemycins A and B in *S*. sp. FXJ1.235.
