# Supplementary material for: Identification and Characterization of Mycemycin Biosynthetic Gene Clusters in Streptomyces olivaceus FXJ8.012 and Streptomyces sp. FXJ1.235
Source: Mar Drugs. 2018 Mar 20;16(3):98. doi: 10.3390/md16030098 (PMC5867642; doi:10.3390/md16030098)
Supplement: Supplementary file 1 [file marinedrugs-16-00098-s001.pdf]

Supplementary Material

Table S1. Genes and proteins involved in mycemycin biosynthesis and their putative functions in *S*. sp. FXJ1.235.

Table S2. Strains used in this study.

Table S3. Plasmids used in this study.

Table S4. Primers used in this study.

Figure S1. HR-ESI-MS spectra of mycemycin A (a) and mycemycin B (b).

Figure S2. UV spectrum of 5-Cl-anthranilic acid.

Figure S3. Proposed pathway for the biosynthesis of mycemycins A and B in *S*. sp. FXJ1.235.

References

Table S1. Genes and proteins involved in mycemycin biosynthesis and their putative functions in *S.* sp. FXJ1.235.

Gene	Size (AA)	Protein Homolog and origin (identity/similarity)	Proposed function
orf-1	47	None predicted in NCBI	Unknown
myeQ	394	KynU, NP_250770.1(47/62); *Pseudomonas aeruginosa* PAO1	Kynureninase
myeC	281	NP_627840.1(86/92); *Streptomyces coelicolor* A3(2)	Tryptophan 2, 3-dioxygenase
orf-2	139	WP_089100086.1(79/86); *Streptomyces hyaluromycini*	DUF3151 domain-containing protein
orf-3	489	WP_059203142.1(88/92); *Streptomyces griseoruber*	MFS transporter
orf-4	27	None predicted in NCBI	Unknown
myeR4	205	SBU95446.1(82/89); *Streptomyces* sp. OspMP-M45	LuxR family transcriptional regulator
myeJ	280	SCE38941.1(73/80); *Streptomyces* sp. PpalLS-921	SAM-dependent methyltransferase
myeR1	334	SBU95411.1(88/92); *Streptomyces* sp. OspMP-M45	Lrp/AsnC family transcriptional regulator
myeK	509	CB02009_orf6, OKJ63402.1(76/84); *Streptomyces* sp. CB02009	Mltidrug MFS transporter
myeR3	676	SBU95407.1(79/84); *Streptomyces* sp. OspMP-M45	SARP family transcriptional regulator
myeR5	226	SBU95417.1(76/83); *Streptomyces* sp. OspMP-M45	TetR family transcriptional regulator
myeP	511	RebH, 4LU6_A (60/72); *Lechevalieria aerocolonigenes*	Tryptophan halogenase
myeO	181	KtzS, ABV56599.1(58/70); *Kutzneria* sp. 744	Flavin reductase
myeN	221	SDU28343.1(51/64); *Amycolatopsis keratiniphila*	Sodium/hydrogen exchanger family
myeA	456	SsfH, ADE34507.1(73/83); *Streptomyces* sp. SF2575	Salicylate synthase
myeG	350	BomK, ALE27503.1 (57/72); *Streptomyces* sp. NRRL 12068	Beta-ketoacyl-ACP synthase (amide bond formation)
myeF	521	PchD, NP_252918.1(49/62); *Pseudomonas aeruginosa* PAO1	2,3-dihydroxybenzoate-AMP ligase
myeE	94	EsmD3, AFB35628.1(55/74); *Streptomyces antibioticus*	Phosphopantetheine-binding protein
myeD	518	KDQ70109.1(84/89); *Streptomyces* sp. NTK 937	Amidohydrolase
orf-5	410	WP_078569189.1(81/86); *Streptomyces*	Phospho-2-dehydro-3-

		sp. NTK 937	deoxyheptonate aldolase
orf-6	256	KFG06189.1(69/81); *Streptomyces scabiei*	2,3-dihydro-2,3-dihydroxybenzoate dehydrogenase

Table S2. Strains used in this study.

Strain	Characteristic	Reference
Escherichea coli		
ETZ12567	*E. coli* host for conjugation	[1]
Top 10	*E. coli* host for cloning	Invitrogen
Rosetta(DE3)	*E. coli* host for heterologous expression	Invitrogen
Rosetta(DE3)/*mymC*	*E.coli* host for MymC heterologous expression	This study
Streptomyces olivaceus FXJ8.012Δ*1741*	Mycemycin C-E producing strain	[2]
Streptomyces sp. FXJ1.235		
FXJ1.235	Mycemycin A-B producing strain	[3]
FXJ1.235ΔmyeP	*ΔmyeP::neo*	This study
FXJ1.235ΔmyeO	*ΔmyeO*	This study
FXJ1.235ΔmyeG	*ΔmyeG::neo*	This study
FXJ1.235ΔmyeD	*ΔmyeD*	This study

Table S3. Plasmids used in this study.

Plasmid	Characteristic	Reference
pET28a	Vector for protein expression, Kan^R	Invitrogen
pET28a::*mymC*	Vector for MymC heterologous expression, Kan^R	
pUC119::*neo*	Source of *neo* (kan^R)	[4]
pKC1139	*E. coli*-*Streptomyces* shuttle vector, Apr^R	[5]
pKC1139::*myeP*::*neo*	*MyeP* deletion construct, Apr^R, Kan^R	This study
pKC1139::*myeO*	*MyeO* deletion construct, Apr^R	This study
pKC1139::*myeG*::*neo*	*MyeG* deletion construct, Apr^R, Kan^R	This study
pKC1139::*myeD*	*MyeD* deletion construct, Apr^R	This study

Apr, apramycin; Kan, kanamycin.

Table S4. Primers used in this study (restriction sites or termini overlaps used are underlined; protective nucleotides are in italics).

Primer	Sequence
Primers for MymC heterologous expression in *E. coli*	
mymC-EF	*G*GAATTCGTGGCACGGCCGGACCGGGACGG
mymC-ER	*CCC*AAGCTTCTACAGGCGGGTGCGGGCGGCCC
Primers for mutant construction and confirmation in FXJ1.235	
myeP-LF	GATCCGCGGCCGCGCGCGATGGACAGGATGGACTGGAAGACG
myeP-LR	GGTATCCAGGGGATAGATCTACATCTTGCGGACGGTGTCTATA
myeP-RF	CTGGGGTTCGGGTAAGATCTGCCCTCCACGTACGACATCCTG
myeP-RR	GACATGATTACGAATTCGATGACTCGTTGCTGCGGATGCTG
ΔmyeP-F	CACCACCGACGGTTTCCTCT
ΔmyeP-R	CCATCAACTGCCGCATCCC
myeO-LF	GATCCGCGGCCGCGCGCGATTGTCCCGTACCAACGCACCACACG
myeO-LR	CGTAGAGCAGAGGGGCGGGGGCCATCAACTGCCGCATCCCGTCG
myeO-RF	CGACGGGATGCGGCAGTTGATGGCCCCCGCCCCTCTGCTCTACG
myeO-RR	GACATGATTACGAATTCGATCCTGGAAGTGGACGCTGCTGTG
ΔmyeO-F	TTCGACTTCACGGTGGACTTCATCC
ΔmyeO-R	ACAGCCACGAACGCCAGTCGC
myeG-LF	GATCCGCGGCCGCGCGCGATCGTGGTCGCGCGGAAGCAC
myeG-LR	GGTATCCAGGGGATAGATCTCGAGATGGATTCCGTCGACCTTCA
myeG-RF	CTGGGGTTCGGGTAAGATCTCCGAGAAGCGGGCTGAGCGATA
myeG-RR	GACATGATTACGAATTCGATCAGGTCGTGCCAGTGGTTGTTGA
ΔmyeG-F	ACAGCATCCGCAGCAACGAGTC
ΔmyeG-R	ACAGGGCGAAGCAGACGATGA
myeD-LF	GATCCGCGGCCGCGCGCGATGAGAAGCGGGGCATCTGGGAG
myeD-LR	GGTATCCAGGGGATAGATCTGTCCGCCGTACAGATGTGTCCG
myeD-RF	CTGGGGTTCGGGTAAGATCTCCGCCCTCTTCGACGACAACCC
myeD-RR	GACATGATTACGAATTCGATACGGGACGGGCTCACGGACCA
ΔmyeD-F	GATGATGCGGCTGGTCAACAAG
ΔmyeD-R	GGAGCGGGGTTTGGCGAACT
neo-*Bgl*II-F	*GA*AGATCTATCCCCTGGATACCGCTCGCCGCAG
neo-*Bgl*II-R	*GA*AGATCTTACCCGAACCCCAGAGTCCCG
T7	TAATACGACTCACTATAGGG
T7ter	GCTAGTTATTGCTCAGCGG

Figure S1. HR-ESI-MS spectra of mycemycins A (a) and mycemycin B (b).

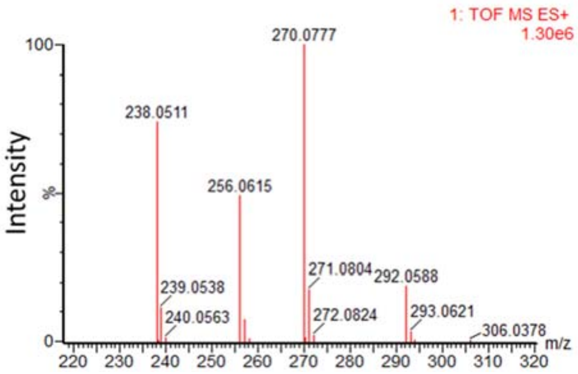

(a)

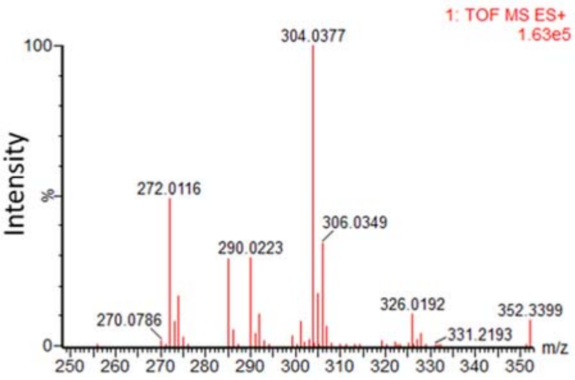

(b)

Figure S2. UV spectrum of 5-Cl-anthranilic acid.

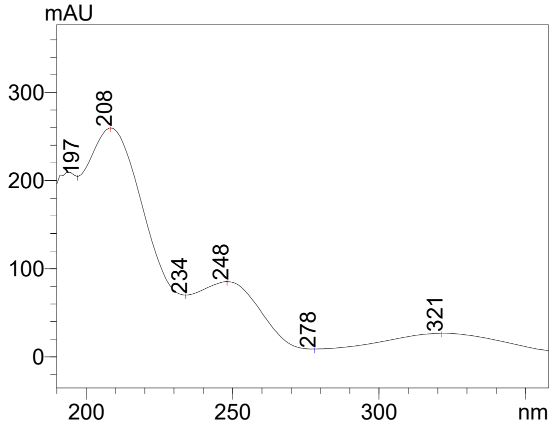

Figure S3. Proposed pathway for the biosynthesis of mycemycins A and B in *S.* sp. FXJ1.235.

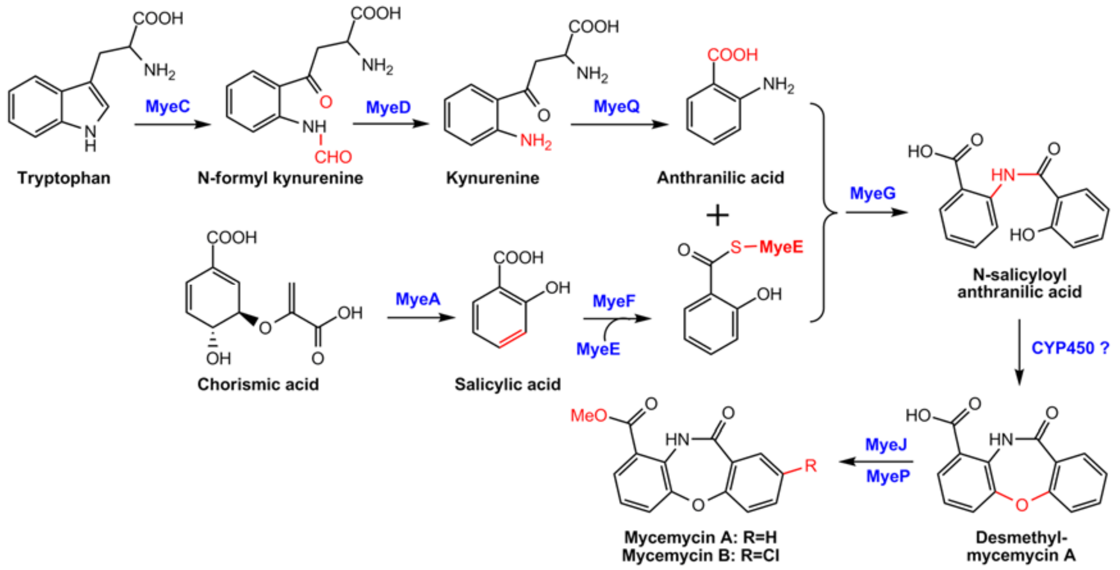

References

1. Macneil, D.J.; Gewain, K.M.; Ruby, C.L.; Dezeny, G.; Gibbons, P.H.; Macneil, T. Analysis of *Streptomyces avermitilis* genes required for avermectin biosynthesis utilizing a novel integration vector. *Gene* **1992,** *111*, 61-68.
2. Liu, N.; Shang, F.; Xi, L.; Huang, Y. Tetroazolemycins A and B, two new oxazole-thiazole siderophores from deep-sea *Streptomyces olivaceus* FXJ8.012. *Mar Drugs* **2013,** *11*, 1524-33.
3. Liu, N.; Song, F.; Shang, F.; Huang, Y. Mycemycins A–E, new dibenzoxazepinones isolated from two different Streptomycetes. *Mar Drugs* **2015,** *13*, 6247-6258.
4. Pan, Y.; Liu, G.; Yang, H.; Tian, Y.; Tan, H. The pleiotropic regulator AdpA-L directly controls the pathway-specific activator of nikkomycin biosynthesis in Streptomyces ansochromogenes. *Mol. Microbiol.* **2009**, *72*, 710–723.
5. Kieser, T.; Bibb, M.J.; Buttner, M.J.; Chater, K.F.; Hopwood, D.A., *Practical Streptomyces Genetics*. John Innes Foundation: Norwich, UK, 2000.
